# Reduced Haematopoietic Output in Automobile Mechanics and Sprayers with Chronic Exposure to Petrochemicals: A Case-Control Study in Cape Coast, Ghana

**DOI:** 10.1155/2018/9563989

**Published:** 2018-03-26

**Authors:** Patrick Adu, Richard Pobee, Aaron Awuah, Paul B. Asiamah, Festus Amoani, Sampson Gyabaa

**Affiliations:** ^1^Medical Laboratory Science Department, School of Allied Health Sciences, University of Cape Coast, Cape Coast, Ghana; ^2^Medical Laboratory Department, Ewim Polyclinic, Cape Coast, Ghana

## Abstract

**Background:**

Automobile mechanics and sprayers are at a higher risk of exposure to hazardous chemicals such as polycyclic aromatic hydrocarbons and heavy metals which may cause adverse health outcomes. This study aimed to use reticulocyte count as an indirect measure of the haematological output in automobile mechanics and sprayers in the Cape Coast Metropolis, Ghana.

**Method:**

This cross-sectional study recruited 130 participants: 90 cases (57 automobile mechanics and 33 automobile sprayers) and 40 controls (nonautomobile workers). Venous blood samples were drawn from the participants and examined for full blood count and absolute reticulocyte count. Semi-structured questionnaire was used to collect demographic and occupational safety information from participants.

**Results:**

75.6% of cases had never received occupational safety training. Whereas 35.1% of automobile mechanics routinely siphoned fuel, 36.4% of automobile sprayers never used nose masks in the discharge of their duties. Controls had significantly higher WBC counts compared to mechanics (*p* = 0.0001; 5.04 ± 1.7 versus 3.81 ± 1.1), or sprayers (*p* = 0.0004; 5.04 ± 1.7 versus 3.74 ± 0.9). Lymphocyte, monocyte, and platelet counts were also significantly higher in controls compared to cases. Whereas RBC counts were significantly higher in controls compared to automobile mechanics (4.85 versus 4.66; *p* = 0.034), haemoglobin levels were significantly higher in automobile sprayers compared to controls (15.13 versus 14.1 g/dl; *p* = 0.0126). Absolute reticulocyte count was significantly higher in controls compared to cases [*p* < 0.0001; (56.88 ± 32.14) × 10^9^/L (controls) versus (25.31 ± 15.75) × 10^9^/L (sprayers) or (33.27 ± 24.42) × 10^9^/L (mechanics)]. Among the cases however, only RBC counts were significantly lower in automobile mechanics compared to automobile sprayers (*p* = 0.0088; 4.66 ± 0.4 versus 4.85 ± 0.5).

**Conclusion:**

It was evident that both automobile mechanics and sprayers had significantly reduced haematopoietic output. Occupational safety training is not given priority and must be addressed.

## 1. Introduction

Exposure to aliphatic and aromatic hydrocarbons has been noted to be deleterious to the health of humankind [1]. As these chemicals are highly volatile and can subsequently be inhaled and/or absorbed through the skin, the potential for these to cause harm may be compounded in regions with high ambient temperature like Ghana. Petrochemicals which also contain some of these aromatic compounds as well as heavy metals consisting of lead, cadmium, chromium, copper, nickel, and vanadium may thus cause adverse health outcomes if exposure to these is not controlled [2].

Certain occupations such as automobile mechanics and sprayers are prone to immediate exposure to these hazardous chemicals through either inhalation, dermal exposure, or ingestion [3]. Their occupational exposure could range from routine transportation, distribution, inhalation of vehicle exhaust fumes, accidental spills, improper handling, and use of petroleum products to leaching of petroleum hydrocarbons [4]. Whereas automobile mechanics may intentionally siphon fuel with their mouth as they try to start the ignition [2], automobile sprayers may indirectly inhale these hazardous chemicals (even with the use of nose masks) when applying a coat (paint, ink, and varnish) unto car surface [5].

Exposure to these aromatic compounds and heavy metals may suppress the bone marrow's activity leading to ineffective hematopoiesis [6]. Heavy metals are noted to affect the haematopoietic system through the inhibition of haemoglobin synthesis by suppressing various key enzymes involved in the heme synthesis pathway and thus lead to reduced life-span of erythrocytes [7]. These aromatic chemicals have been shown to bind to bone marrow DNA leading to chromosomal abnormality [8]. Thus, chronic exposure to these aromatic compounds due to chronic inhalation may lead to undesirable consequence on the haematopoietic system in humans [9]. Although automechanics and automobile sprayers abound in Ghana, there is paucity of data regarding how their routine occupational practices impact their overall health. This study therefore sought to investigate whether the chronic exposure of these automobile workers adversely affected their haematological output. The bone marrow suppressive activity of these aromatic compounds was assessed indirectly through the reticulocyte count and reticulocyte index, which measures the red cell output from the bone marrow [10].

## 2. Materials and Methods

### 2.1. Sampling Sites

This case-control study recruited 130 participants: 90 cases [automobile mechanics (57) and automobile sprayers (33)] and 40 controls at Siwdo in the Cape Coast Metropolis, Ghana. The Siwdo automobile workshop is the largest in the Cape Coast Metropolis. The automobile mechanics included diesel-engine mechanics, motor trucks mechanics, engine repair mechanics, differential and brakes repairers, general automobile-service-station mechanics, workshop heads, and apprentices. A simple convenience sampling technique was employed where all consecutive individuals who gave informed consent were recruited for the study. Questionnaires were also used to collect sociodemographic data and working history of each participant.

### 2.2. Sample Collection and Analysis

About 3-4 mls venous blood was taken into a vacutainer containing K_2_EDTA anticoagulant. Samples were analyzed using automated haematological analyzer (URIT-3000 Plus, China). New methylene blue (NMB) was used to supravitally stain participants' blood samples, after which thin blood film was prepared and used to estimate the reticulocyte counts in accordance with previously published protocols [11]. Microscopically, reticulocytes were recognized by the cytoplasmic presence of NMB-stained ribosomal RNA [11, 12] while counting 1000 red cells.

The percentage of reticulocytes was calculated according to the following formula:

(1) Reticulocyte  count%=number  of  reticulocytesnumber  of  red  cells1000×100%.

Absolute reticulocyte count is a calculated index using the values generated from the reticulocyte count percentage and RBC count from the full blood count. Therefore, the absolute reticulocyte count was calculated for all participants using the following formula:

(2) Absolute  reticulocyte  count=Reticulocyte  count%×RBC  count.

See [12].

### 2.3. Statistical Analysis

GraphPad Prism version 6.0 (GraphPad Software, USA) was used for the data analysis. Data were analyzed for normality using D'Agostino-Pearson omnibus normality test, after which appropriate statistical tests were selected to compare variables. Questionnaires were entered in Microsoft Excel 2010.

## 3. Results

The demographic characteristics of the participants are presented in Table 1. All the participants were males and were mostly in the age group 20–30 years [85% controls versus 66.6% mechanics versus 91.8% sprayers]. A significantly higher proportion of the cases frequently experienced headaches for which they took various analgesic formulations [*p* = 0.0035; 27.5% controls versus 61.4% mechanics versus 54.5% sprayers]. Also, significantly higher proportions of cases had no knowledge on health implications of the frequent handling of petrochemicals and petroleum products [*p* < 0.0001; 70.2% mechanics versus 45.5% sprayers versus 15% controls]. Most of the cases did not use any safety measure in the discharge of their daily duties (74.7% mechanics versus 78.8% sprayers).

The risky occupational-related behavior of the automobile workers is presented in Table 2. A significant proportion of the automobile workers had not received any training on occupational safety (75.6% no versus 24.4% yes; *p* < 0.001). Also, whereas 35.1% of mechanics regularly siphoned fuel in the discharge of their duties, 36.4% of sprayers never used nose mask in their discharge of their duties.

The haematological profiles of the study participants are summarized in Table 3. Mechanics had significantly lower RBC count compared to controls (*p* = 0.0354). Sprayers also had significantly higher haemoglobin levels compared to controls [*p* = 0.0126; 15.13 (sprayers) versus 14.1 g/dl (controls)]. However, whereas the MCH and MCHC were all significantly increased in cases compared to controls, the RDW was significantly reduced in cases compared to controls (*p* < 0.0001 in each case). Additionally, the total WBC counts [*p* = 0.0001 (controls versus mechanics), *p* = 0.0004 (controls versus sprayers)], absolute lymphocyte count [*p* < 0.0001 (control versus mechanics), *p* = 0.0004 (controls versus sprayers)], and MID cell count [*p* < 0.0001 (control versus mechanics), *p* < 0.0001 (controls versus sprayers)] were significantly reduced in cases compared to controls. Moreover, platelet counts were significantly reduced in both mechanics (*p* = 0.0006) and sprayers (*p* = 0.0004) compared to controls.

Among the cases however, only RBC count was significantly higher in sprayers compared to mechanics (*p* = 0.0088, Supplementary Table S1). Additionally, the haematological variables did not differ with respect to years at work in any of the automobile workers (S2 and S3).

**Figure 1:**
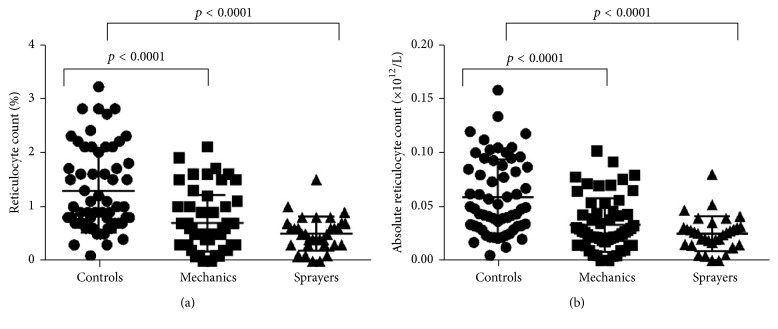
Mean absolute reticulocyte counts of cases (mechanics and sprayers) and controls. Mean absolute reticulocyte count of automobile sprayers and mechanics (cases) as against control group at *p* < 0.05 using ANOVA, Kruskal-Wallis, and Dunn's multiple comparison test.

To understand how the occupational exposure impacted the haematopoietic output, the % reticulocyte count and absolute reticulocyte counts were also compared between cases and controls (Figure 1). The % reticulocyte counts were significantly higher in controls compared to cases [Figure 1(a); *p* < 0.0001 (controls versus mechanics), or *p* < 0.0001 (controls versus sprayers)]. In addition, the absolute reticulocyte counts were significantly higher in cases compared to controls [Figure 1(b); *p* < 0.0001 (controls versus mechanics), *p* < 0.0001 (controls versus sprayers)].

## 4. Discussion

Chronic exposure to petrochemicals may adversely impact haematopoiesis as these contain heavy metals that may be inhibitory to enzymes crucial in blood cell formation [7]. In this study, we sought to investigate the impact of chronic exposure of petroleum products on haematopoietic output in automechanics and automobile sprayers using reticulocyte count as indirect measure of bone marrow erythropoietic output. We found significantly reduced haematopoietic output in automechanics and automobile sprayers as evidenced by generally reduced haematopoietic profiles as well as significantly reduced reticulocyte counts suggesting that exposure to these chemicals may be a potential cause of these adverse haematopoietic functions.

Previously, Aleemuddin et al. suggested that exposure to the heavy metals in aromatic compounds may lead to ineffective erythropoiesis as a consequence of its suppressive activity on the bone marrow [6]. Another cross-sectional study in Pakistan also recorded similar significant reductions in haemoglobin and RBC counts among automobile mechanics and automobile sprayers compared to controls [13]. Although our study did not estimate the levels of these heavy metals in our study participants, our findings are indicative of the fact that the bone marrow activity is negatively affected by exposure to these petrochemicals. Interestingly, significantly higher proportion of the automobile workers were oblivious of the dangers/health implications of the petrochemicals and other products they use in the discharge of their duties. This is in spite of the established role of petrochemicals in suppressing bone marrow activity as well as cause of leukaemia [14, 15]. It is worth noting that majority of our cases complained of frequent work-related headache and body pains for which they regularly took over-the-counter analgesics. Chronic ingestion of these analgesics has also been previously found to cause reduced lymphocyte and neutrophil count [16].

Although some of the findings in the Kamal and Malik [13] study in Pakistan like significantly reduced RBC counts agreed with the findings in this study, the critical findings like significantly increased WBC counts, MCV, and PCV in automobile sprayers compared to controls were at variance with the major findings presented herein. This could be attributed to the fact that majority of the participants in that study were smokers as smoking has been shown to cause increased levels of most haematological parameters. None of the participants in our study responded in the affirmative to smoking. Another study in Nigeria that recruited fuel attendants also reported significantly elevated WBC, granulocytes, lymphocytes, and monocytes in fuel attendants compared to controls [17]. Although the findings presented in this study are at variance with that report [17], it is a feasible supposition that the levels of exposure of automobile mechanics and sprayers to petrochemicals may be higher compared to the fuel attendants. This may be partly responsible for the differing haematological profiles in these two populations. Others that adopted animal model experimentation also reported dose-dependent reduction of haematocrit and haemoglobin concentration in relation to time of exposure [18]. However, among our automobile workers, we could not detect any trend of significant changes in haematopoietic profile with years of work.

It is interesting to note that our cases had significantly reduced red cell distribution width (RDW) compared to our controls. Previous prospective studies as well as meta-analysis have suggested that high RDW was associated with increased risk of cardiovascular disease [19–21]. Although this study did not assess cardiovascular risk of the participants, it is possible that the physical demands of the work of automechanics might offer some protection against adverse cardiovascular effects. As RDW estimates RBC variability that may be increased in reticulocytosis, the significantly reduced reticulocyte counts in our cases could as well have resulted in low RDW. This reduced RDW in our cases should therefore be interpreted in the light of the significantly reduced reticulocyte count. As reticulocyte counts are indirect estimate of the bone marrow haematopoietic/erythropoietic output, it is fairly reasonable to suppose that the chronic exposures of these automobile workers to these petrochemical reagents may have adversely impacted haematopoiesis. Exposure to these aromatic compounds and heavy metals may suppress the bone marrow's activity leading to ineffective hematopoiesis [6] and consequently reduced reticulocyte count. Moreover, judging from the significantly reduced reticulocyte counts in our cases, the apparently normal red cell count and haemoglobin concentration in our cases might have been pseudo as a result of dehydration on the part of our cases. In spite of the high ambient temperature in Ghana, our cases reported taking ≤1.5 L of water during each working day. As plasma is about 70% water, this low fluid intake might have artificially increased the haemoglobin concentration in our cases in spite of their reduced reticulocyte counts.

Risky occupational behavior such as siphoning of fuel into the mouth by automechanics and not wearing nose masks (by automobile sprayers) in the work place was very prevalent in our study population. This is not surprising considering that majority of the cases (automobile sprayers and automechanics) had never received any training in occupational safety. This prevalent practice of aspiration of petrochemicals has previously been reported by others [22, 23]. Although our study revealed suppressed haematopoietic challenges in automobile workers, the impact of our study is limited by our inability to estimate the heavy metal levels in the serum of both our cases and controls. This would have broadened the scope of our findings considerably.

## 5. Conclusions

There is reduced haematopoietic output in automobile mechanics and automobile sprayers. There is also lack of adherence to occupational safety principles which requires urgent attention if long term effects of petrochemical exposures are to be prevented.

## Figures and Tables

**Table 1:** Demographic characteristics of study population.

Variable	Controls (40) *N* (%)	Mechanics (57) *N* (%)	Sprayers (33) *N* (%)	*p* value
*Age (yrs)*				
≤19	5 (12.5)	5 (8.8)	3 (9.1)	
20–39	34 (85)	38 (66.6)	27 (91.8)	
40–59	1 (2.5)	14 (24.6)	3 (9.1)	
*Gender*				
Male	40 (100)	57 (100)	33 (100)	
Female	n/a	n/a	n/a	
*General symptoms Headache *				
Yes	11 (27.5)	35 (61.4)	18 (54.5)	**0.0035**
No	29 (72.5)	22 (38.6)	15 (45.5)	
*Work Experience (yrs)*				
<10	n/a	36 (63.2)	23 (69.7)	
10–20	n/a	14 (24.6)	7 (21.2)	
>20	n/a	7 (12.2)	3 (9.1)	
*Knowledge on any health risk of petrochemicals*				
Yes	34 (85)	17 (29.8)	18 (54.5)	**<0.0001**
No	6 (15)	40 (70.2)	15 (45.5)	

Significant *p* < 0.05; chi-square (*z*) test value; percent out of total; confidence interval 95% CI.

**Table 2:** Occupational safety awareness of participants.

Variable	Yes	No	*p* value
	*N* (%)	*N* (%)	
Have you ever received occupational safety training? *N* = 90	22 (24.4)	68 (75.6)	**<0.001**

Do you siphon fuel with your mouth? (mechanics only; *N* = 57)	20 (35.1)	37 (64.9)	**0.024**

Do you ever wear nose mask? (sprayers only; *N* = 33)	21 (63.6)	12 (36.4)	**0.046**

**Table 3:** Mean hematological parameters among controls and cases.

Parameters	Controls	Mechanics (M)	Sprayers (S)	*p* ^*∗*^	*p* ^*∗∗*^
	*n* = (40)	*n* = (57)	*n* = (33)		
RBC (10^12^/L)	4.85 ± 0.6	4.66 ± 0.4	4.85 ± 0.5	**0.0354**	>0.9999
HGB (g/dL)	14.10 ± 1.6	14.65 ± 1.4	15.13 ± 1.4	0.2659	**0.0126**
HCT%	40.90 ± 5.5	39.86 ± 3.8	41.08 ± 4.2	0.1038	>0.9999
MCV (fL)	83.80 ± 5.9	85.63 ± 5.6	84.91 ± 6.8	0.1030	0.6716
MCH (pg)	28.23 ± 2.6	31.44 ± 2.3	30.67 ± 3.9	**<0.0001**	**<0.0001**
MCHC (g/dL)	34.01 ± 0.7	36.61 ± 1.4	36.79 ± 1.2	**<0.0001**	**<0.0001**
RDW-SD	50.21 ± 4.1	31.33 ± 2.6	30.32 ± 3.1	**<0.0001**	**<0.0001**
WBC (10^9^/L)	5.04 ± 1.7	3.81 ± 1.1	3.74 ± 0.9	**0.0001**	**0.0004**
GRAN # (10^9^/L)	1.76 ± 0.9	1.66 ± 0.7	1.54 ± 0.5	0.8028	0.5187
LYM # (10^9^/L)	2.56 ± 0.7	1.94 ± 0.6	1.99 ± 0.6	**<0.0001**	**0.0004**
MID # (10^9^/L)	0.36 ± 0.16	0.23 ± 0.03	0.224 ± 0.08	**<0.0001**	**<0.0001**
PLT (10^9^/L)	253.1 ± 63.8	210.8 ± 55.1	204.2 ± 48.9	**0.0006**	**0.0004**
MPV (fL)	10.78 ± 0.93	8.77 ± 0.843	8.82 ± 0.871	**<0.0001**	**<0.0001**

ANOVA; Dunn's multiple comparison; *p*^*∗*^: control verses mechanics (M); *p*^*∗∗*^: control verses sprayers (S); results showing mean ± standard deviation at 95% CI confidence interval, significant at *p* < 0.05; GRAN: granulocytes (neutrophils, eosinophils); LYM: lymphocytes; MID: monocytes.

## Abbreviations

RBC: Red cell count
WBC: White blood cell
MCV: Mean cell volume
MCH: Mean cell haemoglobin
MCHC: Mean cell haemoglobin concentration
RDW: Red cell distribution width.

## Supplementary Materials

Supplementary 1 Table S1: comparison of mean haematological parameters between mechanics and sprayers. Respective haematological variables of mechanics and automobile sprayers were compared using Mann–Whitney test. Data are presented as mean ± standard deviation at 95% CI (confidence interval); statistical significance was set at *p* < 0.05.

Supplementary 2 Table S2: comparison of mean haematological parameters of sprayers with varying years of work experience. Haematological variables were compared among automobile sprayers based on years at work. Data are presented as mean ± standard deviation at 95% CI (confidence interval). All statistical comparisons were undertaken using Kruskal-Wallis test with Dunn's post hoc multiple comparison test.

Supplementary 3 Table S3: comparison of mean haematological parameters of mechanics based on years of work experience. Haematological variables were compared among automobile mechanics based on years at work. Data are presented as mean ± standard deviation at 95% CI (confidence interval). All statistical comparisons were undertaken using Kruskal-Wallis test with Dunn's post hoc multiple comparison test.
