# Supplementary material for: Reduced Haematopoietic Output in Automobile Mechanics and Sprayers with Chronic Exposure to Petrochemicals: A Case-Control Study in Cape Coast, Ghana
Source: J Environ Public Health. 2018 Mar 26;2018:9563989. doi: 10.1155/2018/9563989 (PMC5892237; doi:10.1155/2018/9563989)
Supplement: Supplementary 1 — Table S1: comparison of mean haematological parameters between mechanics and sprayers. Respective haematological variables of mechanics and automobile sprayers were compared using Mann–Whitney test. Data are presented as mean ± standard deviation at 95% CI (confidence interval); statistical significance was set at p < 0.05. [file 9563989.f1.docx]

**Supplementary data 1**

**Supplementary table S1: Comparison of mean haematological parameters between mechanics and sprayers**

Respective haematological variables of mechanics and automobile sprayers were compared using Mann-Whitney test. Data are presented as mean ± standard deviation at 95% CI (confidence interval); statistical significance was set at p < 0.05.

| Parameters | | Mechanics (M)  N = (57) | | | Sprayers (S)  N = (33) | | P-value |
| --- | --- | --- | --- | --- | --- | --- | --- |
| WBC (10^9^/L) | | 3.81 ± 1.1 | 3.74 ± 0.9 | | 0.9021 | |  |
| RBC (10^12^/L) | | 4.66 ± 0.4 | 4.85 ± 0.5 | | **0.0088** | |  |
| HGB (g/dL) | | 14.65 ± 1.4 | 15.13 ± 1.4 | | 0.1074 | |  |
| HCT % | | 39.86 ± 3.8 | 41.08 ± 4.2 | | 0.1372 | |  |
| MCV (fL) | | 85.63 ± 5.6 | 84.91 ± 6.8 | | 0.4394 | |  |
| MCH (pg) | | 31.44 ± 2.3 | 30.67 ± 3.9 | | 0.4492 | |  |
| MCHC (g/dl) | | 36.61 ± 1.4 | 36.79 ± 1. | | 0.6631 | |  |
| GRAN# (10^9^/L) | | 1.66 ± 0.7 | 1.54 ± 0.5 | | 0.8072 | |  |
| LYM# (10^9^/L) | | 1.94 ± 0.6 | 1.99 ± 0.6 | | 0.6385 | |  |
| PLT (10^9^/L) | | 210.8 ± 55.1 | 204.2 ± 48.9 | | 0.5924 | |  |

*Respective haematological variables of mechanics and automobile sprayers were compared using Mann-Whitney test. Data are presented as mean ± standard deviation at 95% CI (confidence interval); statistical significance was set at p < 0.05. WBC = white blood cell, RBC = red blood cell, HGB = haemoglobin, HCT = haematocrit, MCV = mean cell volume, MCH = mean corpuscular haemoglobin, MCHC = mean corpuscular haemoglobin concentration, GRAN# = number of granulocyte, LYM# = number of lymphpcyte, PLT = platelet.*
