# Supplementary material for: Reduced Haematopoietic Output in Automobile Mechanics and Sprayers with Chronic Exposure to Petrochemicals: A Case-Control Study in Cape Coast, Ghana
Source: J Environ Public Health. 2018 Mar 26;2018:9563989. doi: 10.1155/2018/9563989 (PMC5892237; doi:10.1155/2018/9563989)
Supplement: Supplementary 2 — Table S2: comparison of mean haematological parameters of sprayers with varying years of work experience. Haematological variables were compared among automobile sprayers based on years at work. Data are presented as mean ± standard deviation at 95% CI (confidence interval). All statistical comparisons were undertaken using Kruskal-Wallis test with Dunn's post hoc multiple comparison test. [file 9563989.f2.docx]

**Supplementary data 2**

**Supplementary table S2:** **Comparison of mean haematological parameters of sprayers with varying years at experience.**

Haematological variables were compared among automobile sprayers based on years at work. Data are presented as mean ± standard deviation at 95% CI (confidence interval). All statistical comparisons were undertaken using Kruskal-Wallis test with Dunn’s post-Hoc multiple comparison test.

|  | Work Experience of Sprayers | | |  |  |  |
| --- | --- | --- | --- | --- | --- | --- |
| Parameter | <10 years | 10 -20years | >20 years | P^x^ | P^y^ | P^z^ |
| WBC (10^9^/L) | 3.80 ± 1.0 | 3.52 ± 0.6 | 3.70 ± 0.9 | ns | ns | ns |
| RBC (10^12^/L) | 4.87 ± 0.5 | 4.92 ± 0.2 | 4.65 ± 0.4 | ns | ns | 0.6803 |
| HGB (g/dL) | 15.04 ± 1.6 | 15.4 ± 0.76 | 15.2 ± 1.1 | ns | ns | ns |
| HCT % | 40.93 ± 4.7 | 42.25 ± 2.8 | 40.25 ± 3.3 | ns | ns | ns |
| MCV (fL) | 84.24 ± 6.8 | 86.22 ± 8.8 | 86.8 ± 3.4 | ns | ns | ns |
| MCH (pg) | 30.13±4.4 | 31.35 ± 2.4 | 32.78 ± 2.3 | ns | 0.6267 | ns |
| MCHC (g/dL) | 36.71 ± 0.9 | 36.47 ± 1.6 | 37.73 ± 1.7 | ns | 0.6841 | 0.6839 |
| GRAN# (10^9^/L) | 1.55 ± 0.6 | 1.50 ± 0.3 | 1.55 ± 0.5 | ns | ns | ns |
| LYM # (10^9^/L) | 2.03 ± 0.6 | 1.85 ± 0.26 | 1.95 ±0.5 | ns | ns | ns |
| PLT (10^9^/L) | 201.4 ± 59.9 | 211.7 ± 37.9 | 159.3 ± 62.3 | ns | 0.8671 | 0.6733 |

*Haematological variables were compared among automobile sprayers based on years at work. P^x^ compared sprayers with <10 years work experience verses sprayers with (10-20) years’ work experience; P^y^ compared sprayers <10 years work experience verses those with >20 years work experience; P^z^ compared sprayers with 10 - 20 years’ work experience verses those with >20 years work experience. (Data are presented as mean ± Standard Deviation); Significant p<0.05; 95% confidence interval (CL).* *All statistical comparisons were undertaken using Kruskal-Wallis test with Dunn’s post-Hoc multiple comparison test.*
