# Supplementary material for: Reduced Haematopoietic Output in Automobile Mechanics and Sprayers with Chronic Exposure to Petrochemicals: A Case-Control Study in Cape Coast, Ghana
Source: J Environ Public Health. 2018 Mar 26;2018:9563989. doi: 10.1155/2018/9563989 (PMC5892237; doi:10.1155/2018/9563989)
Supplement: Supplementary 3 — Table S3: comparison of mean haematological parameters of mechanics based on years of work experience. Haematological variables were compared among automobile mechanics based on years at work. Data are presented as mean ± standard deviation at 95% CI (confidence interval). All statistical comparisons were undertaken using Kruskal-Wallis test with Dunn's post hoc multiple comparison test. [file 9563989.f3.docx]

**Supplementary data 3**

**Supplementary table (S)3:** **Comparison of mean haematological parameters of Mechanics based on years of work experience:**

Haematological variables were compared among automobile mechanics based on years at work. Data are presented as mean ± standard deviation at 95% CI (confidence interval). All statistical comparisons were undertaken using Kruskal-Wallis test with Dunn’s post-Hoc multiple comparison test.

|  | Work Experience of Mechanics | | |  |  |  | |
| --- | --- | --- | --- | --- | --- | --- | --- |
| Parameter | <10 years | 10-20years | >20 years | P^x^ | P^y^ | P^z^ |  |
| WBC(10^9^/L) | 3.84 ± 1.0 | 3.72 ± 1.1 | 3.90 ± 1.8 | ns | ns | ns | |
| RBC(10^12^/L) | 4.68 ± 0.4 | 4.67 ± 0.5 | 4.63 ± 0.5 | ns | ns | ns | |
| HGB(g/dL) | 14.67 ± 1.5 | 14.83 ± 0.9 | 14.53 ± 1.2 | ns | ns | ns | |
| HCT % | 39.99 ± 4.1 | 39.59 ± 2.6 | 39.75 ± 4.7 | ns | ns | ns | |
| MCV(fL) | 85.66 ± 5.7 | 85.40 ± 6.3 | 86.08 ± 2.8 | ns | ns | ns | |
| MCH(pg) | 31.23 ± 2.3 | 31.89 ± 2.7 | 31.52 ± 1.4 | ns | ns | ns | |
| MCHC(g/dL) | 36.44 ± 1.1 | 37.31 ± 1.4 | 35.87 ± 2.1 | 0.2244 | ns | 0.1752 | |
| GRAN#(10^9^/L) | 1.60 ± 0.7 | 1.79 ± 0.8 | 1.73 ± 0.9 | ns | ns | ns | |
| LYM#(10^9^/L) | 2.02 ± 0.6 | 1.75 ± 0.5 | 1.97 ± 0.9 | 0.1957 | ns | ns | |
| PLT(10^9^/L) | 217.2 ± 58.7 | 205.1 ± 51.8 | 193.2 ± 35.4 | ns | 0.8664 | ns | |

*Haematological variables were compared among automobile sprayers based on years at work. P^x^ compared mechanics with <10 years work experience verses mechanics with 10 - 20 years’ work experience; P^y^ compared mechanics with <10 years work experience verses those with >20 years work experience; P^z^ compared mechanics with 10 - 20 years’ work experience verses those with >20 years work experience. Data are presented as mean ± Standard Deviation at 95% confidence interval (CI); Statistical significance was set p<0.05. All statistical comparisons were undertaken using Kruskal-Wallis test with Dunn’s post-Hoc multiple comparison test.*
